# Electromagnetic Shielding Performance of Ta-Doped NiFe_2_O_4_ Composites Reinforced with Chopped Strands for 7–18 GHz Applications

**DOI:** 10.3390/nano15201580

**Published:** 2025-10-16

**Authors:** Mehriban Emek, Ethem İlhan Şahin, Jamal Eldin F. M. Ibrahim, Mesut Kartal

**Affiliations:** 1 Department of Physics, Golbasi Vocational School, Computer Technologies, Adiyaman University, Adıyaman 02040, Turkey; 2 Advanced Technology Research and Application Center, Adana Alparslan Türkeş Science and Technology University, Adana 01250, Turkey; shnethem@gmail.com; 3 Interdisciplinary Research Center for Construction and Building Materials, King Fahd University of Petroleum and Minerals, Dhahran 3261, Saudi Arabia; jamalfadoul@gmail.com; 4 Department of Electronics and Communication Engineering, İstanbul Technical University, Maslak, Istanbul 34469, Turkey; kartalme@itu.edu.tr

**Keywords:** shielding effect, Ta doped NiFe_2_O_4_, mixed oxide, chopped strands, ferrite composites

## Abstract

This study reports the synthesis, structural characterization, and electromagnetic shielding performance of tantalum (Ta)-doped nickel ferrite (NiFe_2_O_4_) composites reinforced with chopped strands. Ta-doped NiFe_2_O_4_ powders were prepared via the conventional mixed-oxide route and sintered at 1200 °C for 4 h, resulting in a well-crystallized single-phase spinel structure. Comprehensive structural and chemical analyses were carried out using X-ray diffraction (XRD), Fourier-transform infrared spectroscopy (FTIR), scanning electron microscopy (SEM), and energy-dispersive X-ray spectroscopy (EDS), confirming the successful incorporation of Ta into the NiFe_2_O_4_ lattice and the uniform microstructural distribution. The ferrite powders were subsequently embedded with chopped strands and epoxy resin through hot pressing to fabricate composites with varying filler contents. The electromagnetic interference (EMI) shielding effectiveness (SE) of the composites was systematically evaluated in the 7–18 GHz frequency range using a network analyzer (NA). The optimized composite, with a thickness of 1.2 mm, demonstrated a maximum SE of 34.74 dB at 17.4 GHz, primarily attributed to interfacial polarization, dipolar relaxation, and multiple scattering effects induced by the chopped strands. The results indicate that the shielding performance of the composites can be precisely tuned by modifying the filler concentration and microstructural characteristics, enabling selective frequency-band applications. Overall, this work highlights the potential of Ta-doped NiFe_2_O_4_/chopped strand composites as lightweight, cost-effective, and high-performance candidates for advanced microwave absorption and electromagnetic shielding applications in defense, and next-generation communication technologies.

## 1. Introduction

The rapid advancement of modern electronics and wireless communication systems has brought electromagnetic (EM) waves, particularly in the gigahertz (GHz) frequency range, into nearly every aspect of daily life [1,2]. High-frequency signals are integral to satellite navigation, radar, 5G communication, and aerospace technologies. However, the increasing density of electronic devices and the miniaturization of components have led to severe problems associated with electromagnetic interference (EMI), often referred to as electromagnetic pollution [3,4]. EMI not only disrupts the performance of sensitive electronic equipment but also poses risks to human health, with studies linking long-term exposure to physiological stress, immune suppression, and neurological effects [5,6,7]. These concerns underscore the urgent need for efficient EMI shielding materials capable of addressing environmental, technological, and health-related challenges [8,9,10].

EMI shielding operates primarily through three mechanisms: reflection, absorption, and multiple internal reflections. Reflection-dominant shielding, often associated with metals and highly conductive fillers, has the drawback of creating secondary EM pollution [11,12]. In contrast, absorption-dominant shielding is increasingly favored because it dissipates EM energy into heat, thereby eliminating harmful secondary radiation [13,14]. The absorption efficiency of a shielding material is closely governed by its dielectric and magnetic losses, polarization effects, conductivity, and impedance matching with free space [15]. Ideal shielding materials must achieve both impedance matching allowing incident waves to penetrate the surface and strong attenuation ensuring rapid dissipation of energy inside the material [16]. Designing such multifunctional materials remains one of the most active areas in EMI research.

Recent advances in EMI shielding have focused on lightweight, flexible, and multifunctional composites [17,18]. Carbon-based nanomaterials such as graphene and carbon nanotubes exhibit excellent conductivity and surface area but often lack sufficient magnetic loss to achieve broadband absorption [19]. Metallic fillers provide high reflection but compromise weight and corrosion resistance [20]. Ferrite-based absorbers, on the other hand, offer a unique advantage due to their intrinsic magnetic losses, chemical stability, and tunable properties via cation doping [21]. Among ferrites, nickel ferrite (NiFe_2_O_4_) spinels are particularly attractive for GHz-range applications owing to their high saturation magnetization, structural stability, and superparamagnetic behavior [22]. They have been extensively investigated for microwave absorption [23], magnetic recording and catalytic applications [24]. However, challenges such as high density and limited impedance matching hinder their widespread use in high-performance EMI shielding [25].

To address these challenges, researchers have employed strategies such as compositing ferrites with polymers [26], introducing dielectric or conducting reinforcements [27], and tailoring their microstructures through chemical doping. Doping with aliovalent cations such as Ta^5+^ offers a promising route to modify the electronic structure, create localized defects, and enhance interfacial polarization, thereby improving dielectric relaxation and absorption capabilities [28]. To date, however, Ta-doped NiFe_2_O_4_ has not been systematically explored for broadband microwave absorption, and no studies have combined it with structural reinforcements such as chopped strands for composite fabrication. This represents a significant gap in the literature.

Structural reinforcement also plays a critical role in composite-based shielding materials. Glass chopped strands, in particular, offer excellent mechanical strength, low density, corrosion resistance, and strong bonding with polymeric matrices. Their incorporation enhances the toughness and structural integrity of composites while providing additional scattering and interfacial polarization sites for EM wave attenuation [29,30]. Chopped-strand-based composites are already utilized in aerospace, marine, and automotive applications due to their lightweight nature and outstanding durability [31,32]. Yet, their integration with advanced ferrite systems for EMI shielding has rarely been investigated.

Several recent reports highlight the limitations of existing EMI shielding systems. For example, NiFe_2_O_4_/epoxy composites have shown SE values around –36 dB at 16.5 GHz for 1.2 mm thickness [18], graphene-based polymer composites typically achieve –30 dB in the 12–18 GHz range [33], and CNT-reinforced systems often require high filler loadings to reach –39 dB at low frequencies [34]. These values, while promising, are insufficient to meet the growing demands for broadband, lightweight, and tunable shielding solutions across radar and communication bands.

In this work, we address these limitations by developing, for the first time, Ta-doped NiFe_2_O_4_/chopped strand composites via the conventional mixed-oxide route followed by hot pressing. The introduction of Ta into NiFe_2_O_4_ enables the stabilization of a single-phase spinel structure, while the incorporation of chopped strands provides lightweight reinforcement, improved interfacial interactions, and additional scattering mechanisms [35]. The composites were thoroughly characterized using XRD, FTIR, SEM and EDS to elucidate their structural, morphological, and chemical properties. Microwave shielding performance was systematically evaluated in the 7–18 GHz frequency range using a network analyzer, covering key communication and radar bands (L, S, C, and X).

Remarkably, a 1.2 mm thick Ta-doped NiFe_2_O_4_/chopped strand composite achieved a minimum shielding effectiveness (SE) of 34.74 dB at 17.4 GHz, surpassing many reported ferrite- and carbon-based systems. The good performance is attributed to the synergistic effects of Ta-induced lattice distortions, enhanced interfacial polarization, and multiple scattering facilitated by chopped strands. These findings establish Ta-doped NiFe_2_O_4_/chopped strand composites as a new class of lightweight, cost-effective, and high-performance EMI shielding materials with significant potential for aerospace, defense, and advanced technologies.

## 2. Materials and Method

### 2.1. Raw Materials and Precursor Synthesis

Nickel ferrite doped with tantalum (Ta–NiFe_2_O_4_) was synthesized using a conventional solid-state route. Analytical-grade oxides were employed: NiO (97%, Acros Organics, Geel, Belgium), Fe_2_O_3_ (99%, Sigma-Aldrich, St. Louis, MO, USA), and Ta_2_O_5_ (99%, Alfa Aesar, Karlsruhe, Germany). The powders were weighed according to the stoichiometric formula of NiFe_2_O_4_ with tantalum substitution and thoroughly mixed in ethanol using a planetary ball mill. Zirconia grinding media were used, and the milling was carried out for 20 h to promote homogeneity and particle size reduction. The obtained suspension was oven-dried at 95 °C for 24 h, followed by calcination at 600 °C for 10 h to initiate solid-state reactions.

The calcined powders were gently ground to achieve a fine particle size distribution and subsequently sintered at 1200 °C for 4 h. Heating and cooling rates were controlled at 120 °C/h to minimize thermal stress. This procedure resulted in a single-phase Ta-doped NiFe_2_O_4_ ferrite suitable for composite fabrication.

### 2.2. Incorporation of Chopped Strands

Commercially available chopped glass strands with an average length of 3–6 mm were supplied by Koloğlu Kimya (Istanbul, Turkey). These strands are known for their high impact resistance, good machinability, and stable mechanical properties. To ensure better interaction with the ferrite phase, the strands were pre-pulverized using an agate mortar before mixing with the sintered Ta doped NiFe_2_O_4_ powder.

Composite batches were prepared using Ta-doped NiFe_2_O_4_ and chopped glass strands at weight ratios of 80:20 and 60:40, respectively. For the 80:20 composition, 1.2 g of Ta-doped NiFe_2_O_4_ (x = 0.01), 0.3 g of chopped strands, and 0.3 g of epoxy resin were combined to produce a 1.8 g composite. For the 60:40 composition, 0.9 g of Ta-doped NiFe_2_O_4_ (x = 0.012), 0.6 g of chopped strands, and 0.3 g of epoxy resin were used to yield the same total mass of 1.8 g composite. The mixtures were placed in sealed plastic containers together with zirconia milling balls and ethanol, and homogenization was performed in a rotary mill for 20 h. After milling, the slurries were dried at 100 °C for 24 h, washed with ethanol by filtration to remove residues, and finally vacuum-dried at 60 °C for another 24 h. These steps yielded homogeneous Ta–NiFe_2_O_4_/chopped strand powders for composite formation.

### 2.3. Composite Fabrication with Epoxy Binder

To consolidate the ferrite–strand mixtures into mechanically stable composites, epoxy resin was employed as a binder. The epoxy content was fixed at a weight ratio of 5:1 (composite powder to epoxy). The blended mixtures were transferred into a steel mold and compacted using a hydraulic press under simultaneous temperature and pressure control. The molding was performed at 100 °C and 5 MPa for 1 h to ensure curing of the epoxy and uniform densification of the samples.

The consolidated composites were prepared in rectangular form with a final thickness of 1.2 mm, optimized for electromagnetic shielding experiments. Two sets of samples corresponding to 80:20 and 60:40 ferrite-to-strand ratios were produced for characterization and testing.

### 2.4. Characterization Techniques

The structural properties of the sintered Ta-doped NiFe_2_O_4_ ferrite and chopped strand composites were investigated using X-ray diffraction (XRD, Bruker D2 Phaser, Karlsruhe, Germany) equipped with Cu-Kα radiation (λ = 1.5406 Å). Data were collected within the 2θ range of 10–70° at a scanning speed of 1°/min. The diffraction patterns were analyzed to identify crystalline phases, confirm the formation of Ta-doped NiFe_2_O_4_, and evaluate the presence of structural features associated with the chopped strands.

Microstructural analysis was performed using a scanning electron microscope (SEM, JEOL JSM-5910LV, Tokyo, Japan) to observe grain morphology and particle distribution characteristics. Elemental composition and dopant incorporation were verified by energy-dispersive X-ray spectroscopy (EDS, Oxford-Inca 7274, Oxford, UK) coupled with the SEM, allowing for semi-quantitative analysis of constituent elements across selected regions.

Fourier transform infrared (FT-IR) spectroscopy was performed using a NICOLET 6700 infrared spectrometer (Madison, WI, USA) to identify the functional groups and bonding characteristics of the samples. Spectra were collected in the range of 4000–500 cm^−1^ at room temperature, in transmittance mode. The Ta-doped NiFe_2_O_4_ powders were placed directly on the sample holder, where they absorbed the incident infrared radiation, enabling the detection of vibrational modes associated with the material’s crystal structure and surface chemistry.

The electromagnetic shielding effectiveness (SE) of the prepared composites was assessed using an N 5230A PNA Series Network Analyzer (Agilent Technologies, Santa Clara, CA, USA) device. Measurements were carried out in the frequency range of 7–18 GHz, encompassing both X- and Ku-band regions. Composite samples with a uniform thickness of 1.2 mm were fitted into a waveguide holder, and the scattering parameters (S_11_ and S_21_) were recorded. Shielding effectiveness values were calculated from the measured transmission and reflection coefficients, enabling the evaluation of absorption- and reflection-dominated mechanisms within the composites.

## 3. Results and Discussion

### 3.1. Phase Formation and Structural Analysis

**Figure 1:**
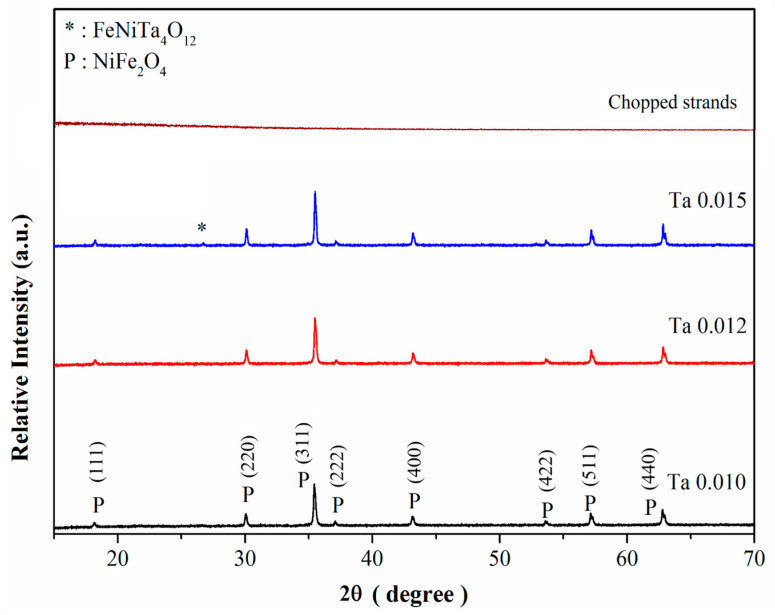
X-ray diffractogram of Ta-doped NiFe_2_O_4_ after 4 h of heat treatment at 1200 °C.

Figure 1 shows the XRD diffractograms of Ta-doped nickel ferrite (Ni_1−x_Ta_x_Fe_2_O_4_) samples with varying Ta contents (x = 0.010–0.015) sintered at 1200 °C for 4 h. The major diffraction peaks observed at 2θ values corresponding to the (111), (220), (311), (222), (400), (422), (511), and (440) planes match well with the standard cubic spinel structure of NiFe_2_O_4_ (JCPDS Card No: 10-0325). At low substitution levels (x = 0.010 and x = 0.012), all reflections are indexed to the cubic spinel phase, confirming the successful incorporation of Ta ions into the lattice without structural distortion. The close similarity between the ionic radii of Ta^5+^ (0.64 Å) and Ni^2+^ (0.69 Å) supports this substitutional mechanism.

With increasing Ta concentration (x ≥ 0.015), additional peaks appear, which are identified as belonging to FeNiTa_4_O_12_ (JCPDS Card No: 60-0552). The intensity of these peaks grows as the Ta content increases, indicating the onset of secondary phase formation. This behavior demonstrates that the solubility limit of Ta in the NiFe_2_O_4_ lattice lies between x = 0.012 and 0.015, beyond which excess Ta segregates from the host spinel phase.

Interestingly, for the composites prepared with Ta-doped NiFe_2_O_4_ and chopped strands, XRD analysis revealed no secondary phases, suggesting that the presence of chopped strands does not disrupt the structural integrity of the ferrite phase. Instead, the ferrite retained its centrosymmetric cubic spinel structure even after composite formation.

### 3.2. Lattice Parameters and Structural Refinement

The diffraction data were further refined using the FullProf software, version 7.80 suite to determine the lattice parameters and unit cell volumes of the doped samples (Ni_1−x_Ta_x_Fe_2_O_4_ (x = 0.010, 0.012, and 0.015)). As summarized in Table 1, the refined values are consistent with those of the cubic spinel symmetry [36], As expected for a cubic spinel structure (space group Fd–3m), the lattice parameters a, b, and c remain equal, and only a slight contraction of the unit cell is observed with increasing Ta substitution. The calculated lattice parameters are approximately 8.3329 Å, 8.3317 Å, and 8.3299 Å for x = 0.010, 0.012, and 0.015, respectively, corresponding to cell volumes of 578.63, 578.37, and 577.99 Å^3^. These marginal reductions in lattice parameter and volume reflect the substitution of smaller Ta^5+^ ions (0.64 Å) for Ni^2+^ ions (0.69 Å) at the octahedral sites, causing a minor lattice distortion while preserving the overall cubic symmetry. The variations are within the experimental uncertainty range of laboratory XRD measurements but are consistent with the expected trend for aliovalent cation substitution. The space group remains Fd–3m (227) across all compositions, confirming that Ta incorporation up to x ≈ 0.012 does not disrupt the spinel framework, while higher Ta concentrations promote the appearance of secondary FeNiTa_4_O_12_ reflections, marking the solubility limit.

### 3.3. Implications for Composite Applications

The confirmation of a stable, centrosymmetric cubic structure at low doping levels is critical for electromagnetic applications. Maintaining single-phase integrity ensures consistent dielectric and magnetic responses, which are essential for impedance matching and absorption in shielding composites [37]. The results highlight that doping concentrations at or below x = 0.012 offer the best structural stability, while higher concentrations introduce phase impurities that may compromise electromagnetic performance.

### 3.4. Microstructural Analysis (SEM)

**Figure 2:**
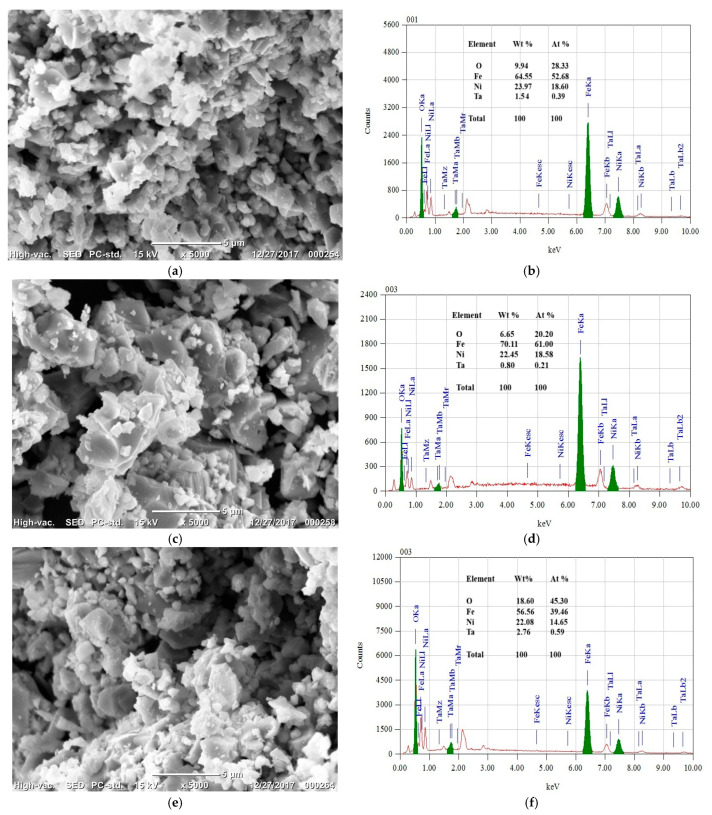
SEM photographs of single-phase Ta-doped Ni_1−x_Ta_x_Fe_2_O_4_ heat treated for 4 h at 1200 °C (**a**) x = 0.01 at ×5000; (**b**) EDS examination of Ta-doped Ni_1−x_Ta_x_Fe_2_O_4_ at ×5000 for x = 0.01; (**c**) x = 0.012 at ×5000; (**d**) EDS examination of Ta-doped Ni_1−x_Ta_x_Fe_2_O_4_ at ×5000 for x = 0.012 and (**e**) x = 0.015 at ×5000; (**f**) EDS examination of Ta-doped Ni_1−x_Ta_x_Fe_2_O_4_ at ×5000 for x = 0.015.

The surface morphologies of Ta-doped NiFe_2_O_4_ ceramics sintered at 1200 °C for 4 h were examined using SEM (Figure 2a,c,e). At low Ta concentrations (x = 0.01), the samples exhibited a relatively loose and porous microstructure, with clearly visible pores distributed throughout the surface, consistent with the centrosymmetric cubic spinel structure confirmed by XRD. No evidence of microstructural impurities or secondary phases was observed. This indicates successful substitution of Ta into the NiFe_2_O_4_ lattice without destabilizing the spinel phase.

With increasing Ta concentration to x = 0.012 (Figure 2c) the grain size becomes noticeably larger, and the grains appear more distinct, while the pore size also increases. This suggests that Ta addition enhances grain growth but simultaneously prevent densification, possibly due to the segregation of Ta ions at grain boundaries, which limits mass transport during sintering. At x = 0.015 (Figure 2e), the microstructure exhibits relatively larger pore sizes and coarser grains, likely resulting from secondary phase formation and partial melting effects at elevated dopant levels.

### 3.5. Compositional Analysis (EDS)

Elemental compositions were investigated using energy-dispersive spectroscopy (EDS) on the powder samples (Figure 2b,d,f). For samples with x = 0.01 and x = 0.012, the EDS spectra closely matched the theoretical stoichiometry of NiFe_2_O_4_, with measured atomic percentages of approximately 23.65% O, 51.76% Fe, and 24.59% Ni. Quantitative EDS analysis confirmed the presence of Ta with measured atomic percentages of approximately 0.95 at.% for (x = 0.010), 1.13 at.% for (x = 0.012), and 1.41 at.% for (x = 0.015), aligning closely with the nominal doping values. These results confirm the successful incorporation of Ta into the spinel lattice without phase segregation at lower doping levels.

With increasing Ta content, however, EDS revealed a proportional increase in the concentration of secondary phases, correlating with the additional peaks detected in the XRD patterns and the microstructural evidence from SEM. These results further reinforce the conclusion that the solubility limit of Ta in NiFe_2_O_4_ lies between x = 0.012 and x = 0.015.

### 3.6. Implications for Electromagnetic Shielding

The microstructural analysis of Ta-doped NiFe_2_O_4_ revealed a loose and porous grain distribution with partially connected grain boundaries, which play a crucial role in enhancing the material’s electromagnetic shielding performance. Grain boundaries and interfacial regions act as scattering centers, enhancing multiple internal reflections of incident electromagnetic waves. Additionally, the large specific surface area and porous features of the sintered microstructure increase interfacial polarization, thereby promoting absorption-dominated shielding.

Taken together, SEM and EDS analyses confirm that maintaining Ta doping at or below x = 0.012 ensures the formation of a single-phase cubic spinel structure with favorable microstructural features for electromagnetic shielding. Doping levels beyond this threshold result in secondary phase formation, which may alter the dielectric and magnetic responses, potentially diminishing shielding efficiency [38,39].

**Figure 3:**
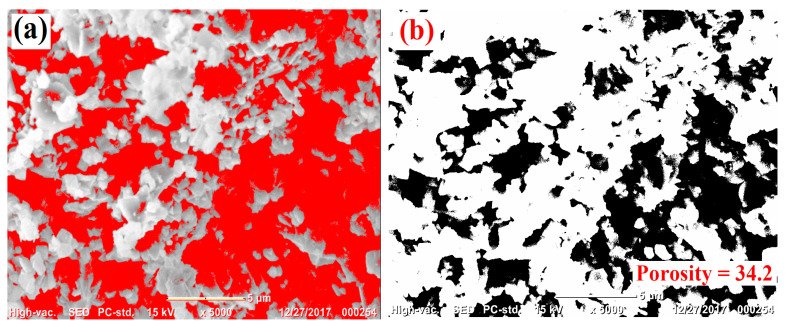

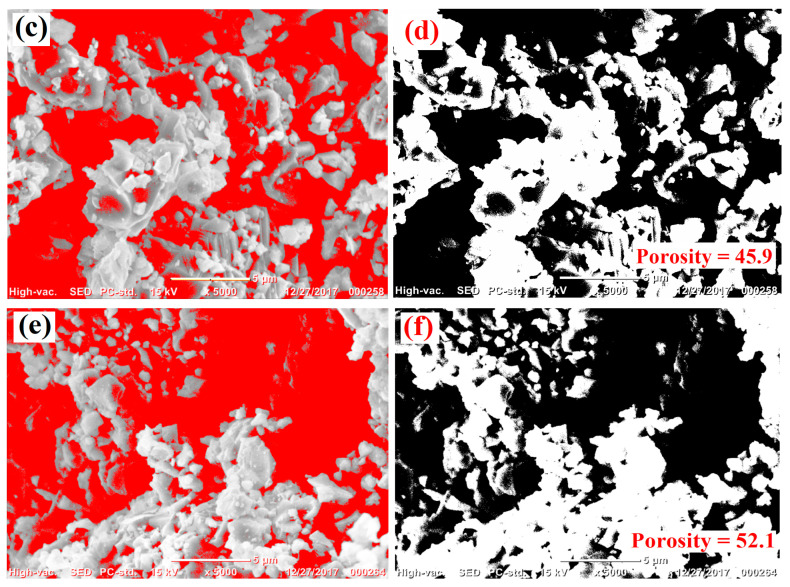
SEM image of single-phase Ta-doped Ni_1−x_Ta_x_Fe_2_O_4_ heat treated for 4 h at 1200 °C showing different pore densities, examined via ImageJ 1.53m software; (**a**) Ta-doped Ni_1−x_Ta_x_Fe_2_O_4_, x = 0.01; (**b**) Ta-doped Ni_1−x_Ta_x_Fe_2_O_4_, x = 0.01 with only pores shown; (**c**) Ta-doped Ni_1−x_Ta_x_Fe_2_O_4_, x = 0.012; (**d**) Ta-doped Ni_1−x_Ta_x_Fe_2_O_4_, x = 0.012 with only pores shown;(**e**) Ta-doped Ni_1−x_Ta_x_Fe_2_O_4_, x = 0.015; (**f**) Ta-doped Ni_1−x_Ta_x_Fe_2_O_4_, x = 0.015 with only pores shown.

The pore characteristics of the ceramic samples were quantitatively evaluated using ImageJ software 1.53m, which enabled image-based porosity measurements (Figure 3). For this purpose, scanning electron microscopy (SEM) micrographs captured at 5000× magnification were processed. The grayscale intensity of each SEM image was adjusted and converted into binary form, allowing clear separation between pore regions and the ceramic matrix. In these processed images, the pore areas appeared as red and black zones, while the solid body of the sample was represented in grey. The calculated average porosity values for Ta-substituted Ni_1−x_Ta_x_Fe_2_O_4_ at x = 0.01, 0.012, and 0.015 were 24.2%, 45.9%, and 52.1%, respectively, indicating a progressive rise in porosity with higher Ta incorporation. With respect to shape, the pores were mainly irregular in geometry, dominating the overall pore network.

### 3.7. FTIR Measurements of Ta Doped NiFe_2_O_4_

**Figure 4:**
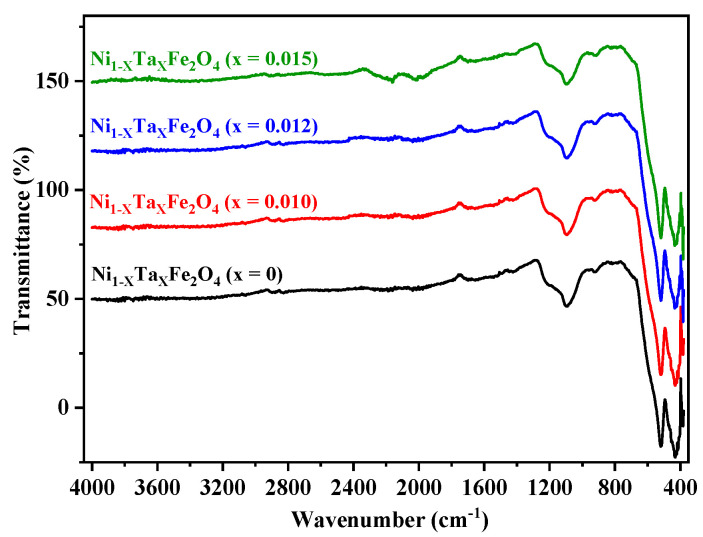
FTIR spectrum of Ta-doped NiFe_2_O_4_ (x = 0, 0.01, 0.012 and 0.015) heat-treated at 1200 °C for 4 h.

The FTIR spectrum of Ta-doped NiFe_2_O_4_ was recorded in the frequency range of 4000–500 cm^−1^ to examine the structural features and vibrational dynamics of the material (Figure 4). A noticeable absorption band observed at ~1100 cm^−1^ is attributed to stretching vibrations associated with metal–oxygen–metal linkages and possible contributions from Ta–O bonds, indicating the successful incorporation of Ta into the spinel lattice. Additionally, a distinct peak at ~900 cm^−1^ corresponds to the deformation vibration of Fe–OH groups, reflecting surface hydroxyl functionalities, which are typically related to residual adsorbed moisture or lattice hydroxyl groups [40].

Below 600 cm^−1^, the spectrum is dominated by characteristic metal–oxygen (M–O) vibrations, which are the signature features of the spinel ferrite structure. Spinel ferrites, including NiFe_2_O_4_, exhibit two principal absorption regions corresponding to the stretching vibrations of M–O bonds at tetrahedral (A-site) and octahedral (B-site) positions. In the present system, Fe^3+^ ions occupy both tetrahedral and octahedral sites, while Ni^2+^ ions are predominantly located in the octahedral coordination environment. At the tetrahedral site, Fe^3+^ ions are coordinated with four oxygen atoms, forming AO_4_ units, whereas Ni^2+^ and Fe^3+^ ions in the octahedral site are coordinated by six oxygen atoms, forming BO_6_ units [41].

Typically, the higher-frequency band in the <1000 cm^−1^ region corresponds to tetrahedral M–O stretching vibrations, while the lower-frequency band originates from octahedral M–O stretching vibrations. These well-defined absorption features confirm the retention of the cubic spinel framework in the Ta-doped NiFe_2_O_4_ material. The incorporation of Ta^5+^ ions, which possess a larger ionic radius and higher charge compared to Fe^3+^, can influence the local bonding environment, leading to subtle band shifts and the emergence of additional features such as the ~1100 cm^−1^ absorption band. This observation strongly supports the substitutional role of Ta in the NiFe_2_O_4_ lattice.

### 3.8. Magnetic Properties of Ta Doped NiFe_2_O_4_ Composites

**Figure 5:**
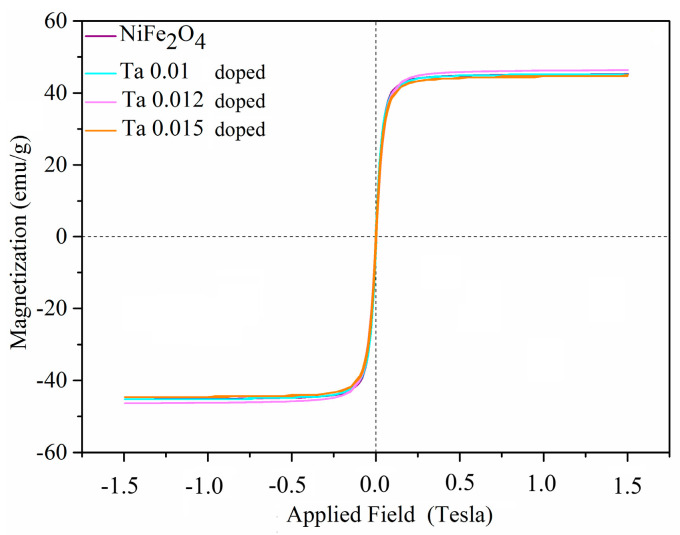
Ta-doped NiFe_2_O_4_ (x = 0.01, 0.012, 0.015) magnetic hysteresis curves after 4 h of heat treatment at 1200 °C.

The magnetic behavior of Ta-doped NiFe_2_O_4_ samples sintered at 1200 °C was investigated at room temperature, and the corresponding hysteresis loops are presented in Figure 5. All compositions exhibited typical soft ferrimagnetic behavior, characterized by narrow hysteresis loops and well-defined saturation magnetization (Ms), indicating their suitability for high-frequency applications.

For the Ni_1−x_Ta_x_Fe_2_O_4_ system, a progressive variation in Ms was observed with increasing Ta content. At a doping level of x = 0.01, the sample exhibited a saturation magnetization of 45.3 emu/g. A further increase in dopant concentration to x = 0.012 resulted in an enhanced Ms value of 46.3 emu/g. This improvement can be attributed to the partial substitution of Ni^2+^ ions by Ta^5+^ ions at the octahedral (B) sites, leading to a redistribution of cations between the tetrahedral (A) and octahedral (B) sublattices. Such redistribution modifies the superexchange interactions between Fe^3+^ ions at the A- and B-sites, thereby enhancing net magnetization [42].

However, at x = 0.015, a slight decrease in Ms (≈ 45.0 emu/g) was observed. This reduction correlates with the emergence of secondary phases, as confirmed by XRD analysis, specifically the FeNiTa_4_O_12_ phase. The presence of these nonmagnetic or weakly magnetic phases dilutes the overall ferrimagnetic ordering, thereby lowering the effective magnetization.

The remanent magnetization (Ms) values for the Ni_1−x_Ta_x_Fe_2_O_4_ samples were observed to be 5.2, 5.4, and 5.1 emu/g for doping concentrations of x = 0.010, 0.012, and 0.015, respectively.

Coercivity (Hc) values remained relatively small across all doping concentrations, consistent with soft ferrimagnetic characteristics [43]. The observed low coercivity coupled with moderate saturation magnetization is advantageous for microwave absorption and EMI shielding applications, as it facilitates rapid domain wall motion under alternating fields [44].

The optimal Ta doping level was determined at x = 0.012, where a balance between structural stability and enhanced magnetic interactions yields the highest Ms value. The decrease at higher dopant concentrations highlights the importance of controlling Ta solubility in the NiFe_2_O_4_ lattice to prevent secondary phase formation [45].

### 3.9. EMI Shielding Measurements of Ta Doped NiFe_2_O_4_/Chopped Strands Composites

The shielding effectiveness (SE) of Ta-doped NiFe_2_O_4_/chopped strands composites embedded in epoxy was investigated in the frequency range of 7–18 GHz using a network analyzer (N523A PNA series). The experimental setup ensured a consistent 50 Ω impedance at both ports through a coaxial sample holder system, and each rectangular composite sample with thickness: 1.2 mm and dimensions 3.5 cm × 1.5 cm × 0.12 cm was placed directly in the NA test fixture and carefully positioned under uniform pressure to guarantee reliable and reproducible results.

The SE values were calculated by comparing transmitted signals with and without the sample in the test fixture. The total shielding effectiveness (SE_T_) was decomposed into contributions from reflection (SE_R_) and absorption (SE_A_) (Equation (1)):

*S*E_T_ = SE_R_ + SE_A_ (1)

where SE_R_ arises from impedance mismatch at the surface, and SE_A_ represents the material’s absorption of electromagnetic energy. These were calculated from the NA S-parameters (S_11_) and (S_21_) Equation (2).

(2) $${SE}_{T}=10\;log10\frac{\left(P\;in\;\right)}{\left(P\;out\right)}=-10\;log10\;{\left|S_{21}\right|}^{2}$$

where (P in) and (P out) represent the incident and transmitted electromagnetic powers, respectively.

Shielding effectiveness increases with sample thickness. To allow direct comparison across different thicknesses, the normalized SE per unit thickness (dB/mm) was calculated Equation (3):

SE_norm_ = SE_T_/t (3)

where (t) is the sample thickness in mm.

**Figure 6:**
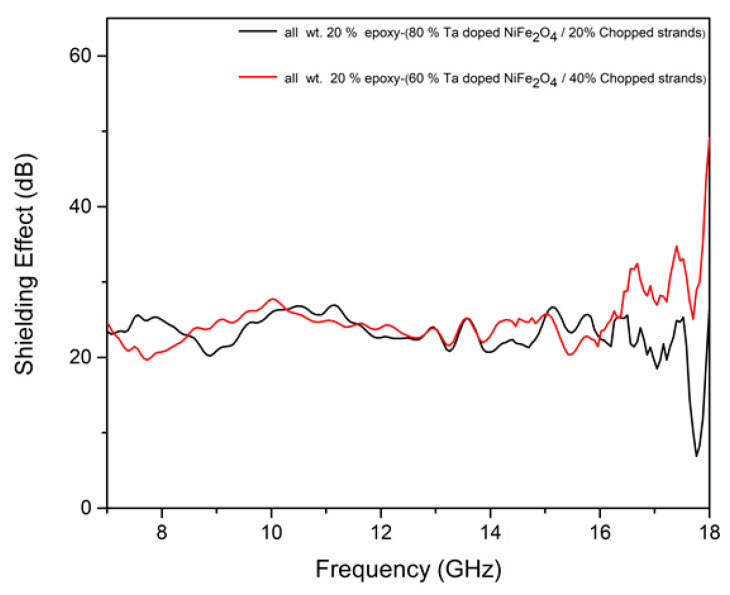
Shielding effect of the epoxy-(Ta doped NiFe_2_O_4_/chopped strands) composites: all wt. 20% epoxy-(80% Ta doped NiFe_2_O_4_/20% Chopped strands) compositions, all wt. 20% epoxy-(60% Ta doped NiFe_2_O_4_/40% Chopped strands) compositions.

Figure 6 illustrates the frequency-dependent variation in SE for the composites with weight ratios of 60–40% and 80–20% (Ta-doped NiFe_2_O_4_ to chopped strands). Both composites demonstrated wideband shielding performance, with SE values consistently below –10 dB across the entire X- and Ku-band region (7–18 GHz), confirming their effectiveness as electromagnetic interference (EMI) shielding materials.

In this study, the measurements presented in Figure 6 and Table 2 were obtained using a network analyzer operating in peak detection mode. Each data point represents a single measurement for a given sample. It should be noted that the values reported are subject to the inherent measurement noise and uncertainty of the instrument, and are not “best values” derived from multiple repeated measurements. Accordingly, the fractional GHz differences should be interpreted as approximate values within the resolution and accuracy limits of the network analyzer.

The shielding effectiveness (SE) values reported in this study were calculated from the measured S-parameters using the standard relation (Equation (2)). Although the raw data yield negative dB values indicating signal attenuation, SE is conventionally reported as a positive quantity representing the magnitude of attenuation; hence, all SE values in this work are expressed as positive dB.

For the 60–40% composite, strong and consistent shielding performance was observed across the measured frequency range. The average SE value was 25.02 dB, confirming its effective broadband shielding capability. The sample exhibited a maximum SE of 34.74 dB at 17.4 GHz, along with notable attenuation peaks at 21.57 dB (7.48 GHz), 25.23 dB (13.55 GHz), 26.33 dB (16.26 GHz), and 31.77 dB (16.57 GHz). These results demonstrate that the 60–40% composite maintains efficient shielding across a wide frequency range.

For the 80–20% composite, consistent shielding behavior was observed across the measured frequency range. The average shielding effectiveness (SE) over the entire 7–18 GHz range was 23.26 dB, demonstrating strong overall attenuation performance. The composite achieved a maximum SE of 27.07 dB near 11.14 GHz, with additional high SE values of 25.73 dB (7.5 GHz), 27.07 dB (11.14 GHz), 25.37 dB (16.37 GHz), and 21.87 dB (17.17 GHz). While SE values fluctuated across the band, the relatively high average SE confirms that the 80–20% composite maintains effective broadband shielding comparable to the 60–40% sample.

The higher shielding performance of the 60–40% composite can be attributed to the synergistic role of chopped strands in enhancing impedance matching and creating additional interfaces for multiple reflections and scattering of incident microwaves. The porous microstructure of Ta-doped NiFe_2_O_4_, combined with the large surface area of chopped strands, promotes interfacial polarization and grain boundary scattering, thereby improving absorption-dominated shielding. Furthermore, the uniform dispersion of chopped strands within the ferrite–epoxy matrix aids in suppressing electromagnetic transmission by balancing reflection and absorption contributions.

Overall, both composites demonstrate efficient EMI shielding across the 7–18 GHz band, with the 60–40% system showing superior broadband attenuation. The results highlight the tunability of shielding performance through composition control, indicating that optimized ratios of Ta-doped NiFe_2_O_4_ and chopped strands can be tailored for specific microwave applications.

The observed variation in magnetic and shielding properties can also be correlated with the possible formation of antisite defects within the Ta-doped NiFe_2_O_4_ spinel lattice. During high-temperature sintering, partial substitution of Ta^5+^ for Ni^2+^ at the octahedral (B) sites may induce local charge imbalance and promote cation redistribution between tetrahedral (A) and octahedral (B) positions, leading to cation inversion. Such antisite disorder, as reported in related spinel systems [46,47], modifies the Fe^3+^–O^2−^–Fe^3+^ and Fe^3+^–O^2−^–Ni^2+^ superexchange interactions, thereby influencing the net magnetization and electronic transport behavior. In our samples, the enhancement in saturation magnetization at x = 0.012 and the corresponding improvement in absorption-dominated EMI shielding (37.74 dB) can be attributed, at least in part, to these defect-induced effects. The presence of antisite defects facilitates electron hopping between Fe^2+^/Fe^3+^ pairs, enhances dielectric loss through localized polarization, and increases magnetic permeability, all of which contribute to improved electromagnetic absorption and overall shielding efficiency.

## 4. Conclusions

In this study, Ta-doped NiFe_2_O_4_–chopped strand composites were successfully synthesized and characterized, and their electromagnetic shielding performance was evaluated in the 7–18 GHz frequency range. The results confirm that the conventional mixed-oxide route effectively produces a well-crystallized, single-phase spinel structure of NiFe_2_O_4_ when doped with tantalum at x = 0.010 and x = 0.012. The solubility limit of Ta in the NiFe_2_O_4_ lattice was identified below x = 0.015, as higher doping levels resulted in the formation of secondary phases such as FeNiTa_4_O_12_. Structural and chemical analyses (XRD, FTIR, SEM, and EDS) verified the successful incorporation of Ta and the homogeneous distribution of the ferrite and chopped strand phases.

The composites fabricated with 80–20% and 60–40% weight ratios of Ta-doped NiFe_2_O_4_ to chopped strands exhibited strong microwave shielding performance. The epoxy-based composite containing 60–40% Ta-doped NiFe_2_O_4_–chopped strands achieved an average shielding effectiveness (SE) of 34.74 dB at 17.4 GHz, indicating efficient broadband attenuation primarily governed by absorption. This enhanced performance is attributed to the synergistic influence of Ta-induced lattice distortions, interfacial polarization, and multiple scattering facilitated by the chopped strands.

Overall, the Ta-doped NiFe_2_O_4_–chopped strand composites demonstrate promising potential as lightweight, cost-effective, and efficient electromagnetic shielding materials for aerospace, defense, and next-generation communication technologies. Future work will focus on incorporating quantitative mechanism analysis, comparative benchmarking with existing systems, and optimizing the dopant concentration and composite architecture to further enhance absorption efficiency and broadband performance.

## Figures and Tables

**Table 1:** Lattice parameters of a NiFe_2_O_4_ specimen were calculated using FullProf.

Sample	a (Å)	b (Å)	c (Å)	Volume (Å^3^)	Space Group
NiFe_2_O_4_	8.3390	8.3390	8.3390	579.950	Fd-3m (227)
Ni_1−x_Ta_x_Fe_2_O_4_(x = 0.010)	8.3329	8.3329	8.3329	578.6259	Fd-3m (227)
Ni_1−x_Ta_x_Fe_2_O_4_(x = 0.012)	8.3317	8.3317	8.3317	578.3739	Fd-3m (227)
Ni_1−x_Ta_x_Fe_2_O_4_(x = 0.015)	8.3299	8.3299	8.3299	577.9970	Fd-3m (227)

**Table 2:** The shielding effect values of the generated samples at various frequencies.

Sample	SE (dB)	Frequency (GHz)
All wt. 20% epoxy-(60% Ta doped NiFe_2_O_4_/40% chopped strands)	21.57	7.48
	24.09	8.63
	25.21	9.08
	26.19	9.56
	27.75	10.02
	24.92	11.06
	24.48	11.62
	24.33	12.12
	23.96	12.96
	25.23	13.55
	25.82	15.01
	22.91	15.74
	26.33	16.26
	31.77	16.57
	32.58	16.68
	29.61	16.91
	28.27	17.10
	34.74	17.40
	33.01	17.51
	10	7–18
	20	7–18
	30	16.57–16.76
		17.24–17.59
All wt. 20% epoxy-(80% Ta doped NiFe_2_O_4_/20% chopped strands)	25.73	7.5
	27.52	7.88
	24.63	9.58
	27.07	11.14
	24.09	12.95
	25.14	13.58
	22.47	14.39
	26.64	15.14
	25.81	15.76
	25.37	16.37
	24.03	16.74
	21.35	16.92
	21.87	17.17
	25.29	17.51
	10	7–17.5
	20	7–17.6

## Data Availability

The original contributions presented in this study are included in the article. Further inquiries can be directed to the corresponding author.
